# CAR-M Therapy: From Concept to Clinical Translation in Solid Tumors

**DOI:** 10.3390/cells15121113

**Published:** 2026-06-19

**Authors:** Chenxi Miao, Zhitao Chen, Juan Chen, Jiazeng Sun, Yanan Sun, Wenbiao Shi, Wentao Xu, Yixuan Li, Xingwang Zhao

**Affiliations:** Key Laboratory of Precision Nutrition and Food Quality, Department of Nutrition and Health, China Agricultural University, Beijing 100083, China; miaochenxi272526@163.com (C.M.); 15007141169@163.com (Z.C.); chenjuan@cau.edu.cn (J.C.); sunjiazeng331@163.com (J.S.); 15153515695@163.com (Y.S.); wenbiao.shi@cau.edu.cn (W.S.); xuwentao@cau.edu.cn (W.X.)

**Keywords:** CAR-macrophage, solid tumors, tumor microenvironment, adoptive cell therapy, phagocytosis, epitope spreading, clinical translation

## Abstract

While chimeric antigen receptor (CAR)-T-cell therapies have shown significant effectiveness in hematological malignancies, their efficacy in solid tumors remains limited by the hostile tumor microenvironment (TME) and antigen heterogeneity. Recently, CAR-Macrophage (CAR-M) therapy has emerged as a paradigm-shifting approach, leveraging the innate capability of macrophages to deeply infiltrate tumors and their plasticity to reverse immunosuppression. Unlike T cells, CAR-Ms not only mediate direct phagocytosis but also initiate epitope spreading, effectively bridging innate and adaptive immunity. This review critically examines the trajectory of CAR-M therapy from biological rationale to clinical reality. We dissect the engineering evolution of CAR constructs, arguing for macrophage-specific signaling domains (e.g., FcRγ, Megf10) over traditional T-cell designs. Crucially, we address the major bottlenecks in clinical translation, including the manufacturing challenges of non-expanding primary macrophages and the emerging shift toward induced pluripotent stem cell (iPSC)-derived platforms. Furthermore, we evaluate current clinical trial landscapes and discuss next-generation strategies such as in vivo programming via lipid nanoparticles (LNPs) and synthetic logic-gating to enhance safety. Ultimately, overcoming manufacturing constraints and optimizing delivery systems will be pivotal for CAR-M to evolve from a niche therapy into a standard-of-care modality for solid tumors.

## 1. Introduction

Despite the transformative success of chimeric antigen receptor (CAR) T-cell therapy in hematologic malignancies, its efficacy against solid tumors remains severely constrained [1]. This limitation is primarily orchestrated by the hostile tumor microenvironment (TME). Unlike systemic circulation, solid tumors are fortified by a dense extracellular matrix (ECM) and aberrant vasculature, establishing a physical fortress that actively thwarts T-cell infiltration [2,3]. Furthermore, the TME imposes a “metabolic desert”—characterized by nutrient deprivation and chronic hypoxia—which precipitates T-cell exhaustion and anergy [4,5,6]. Consequently, conventional CAR-T cells often fail to penetrate these desmoplastic barriers or sustain robust cytotoxicity, underscoring the imperative for therapeutic vehicles with intrinsic TME-homing capabilities [7,8,9].

In this landscape, macrophages emerge as formidable candidates. As the most abundant immune infiltrates within the TME, often comprising up to 50% of the tumor mass, macrophages possess an innate capacity to traverse the physiological barriers that impede T cells [10,11]. Although tumor-associated macrophages (TAMs) frequently adopt a pro-tumorigenic M2 phenotype to facilitate angiogenesis and immunosuppression, they retain remarkable functional plasticity [12]. This biological versatility provides a strategic therapeutic window: rather than focusing solely on TAM depletion, genetic engineering can “repurpose” their potent phagocytic and antigen-presenting machinery. By tethering these cells to CARs, these erstwhile “protumoral agents” can be reprogrammed into potent effectors that not only execute direct phagocytosis but also catalyze a broader adaptive immune response via epitope spreading [13,14].

The concept of redirecting myeloid cells was pioneered by Biglari et al. using CD64-based monocytes [15], but recent advances in viral transduction efficiency have propelled the field toward clinical realization. Currently, pioneering CAR-M therapies, such as CT-0508 (anti-HER2) and MCY-M11 (anti-Mesothelin), have secured FDA Investigational New Drug (IND) clearance and entered Phase I trials [16]. Distinct from CAR-T cells, CAR-Ms leverage dual mechanisms—targeted phagocytosis and holistic TME remodeling—to tackle solid tumors. Nevertheless, widespread clinical translation is hindered by several bottlenecks: (1) manufacturing complexity, particularly the non-proliferative nature of mature macrophages; (2) suboptimal in vivo persistence; and (3) the exigency of ensuring precise trafficking while mitigating systemic toxicity [10,17].

This review provides a systematic dissection of the CAR-M trajectory, from fundamental biological principles to clinical translation. We specifically evaluate: (1) The engineering evolution, moving beyond the “generations” concept of CAR-T to specific screening of macrophage-tailored signaling domains (e.g., FcRγ, Megf10); (2) mechanistic insights into how CAR-Ms bridge innate and adaptive immunity; and (3) Translational barriers, providing a critical analysis of cell sourcing (PBMC vs. iPSC) and manufacturing scalability. Finally, we discuss next-generation strategies, such as in situ reprogramming, aimed at redefining the landscape of solid tumor immunotherapy.

## 2. Biological Rationale: Why Macrophages Are Ideal Candidates

Despite the transformative success of CAR-T therapy in hematologic malignancies, its efficacy in solid tumors remains severely constrained by the hostile TME. The dense ECM and aberrant vasculature establish a physical fortress that actively thwarts T-cell infiltration, while the metabolic landscape precipitates T-cell exhaustion. Within this framework, macrophages emerge not merely as alternative cellular vehicles, but as biologically superior candidates uniquely equipped to penetrate and remodel the solid tumor architecture.

### 2.1. Active Homing and Intratumoral Infiltration

Although T cells can actively infiltrate tumors through chemokine-directed migration and adhesion-mediated extravasation, their accumulation is often restricted by abnormal tumor vasculature and dense stromal barriers. In contrast, macrophages possess an intrinsic ability to navigate and colonize solid tumors, including poorly vascularized and hypoxic regions. This recruitment is actively driven by the tumor’s own chemokine network [18]. Solid tumors secrete high levels of chemokines, such as CCL2 (MCP-1) and CSF-1, to recruit circulating monocytes via the CCR2-CCL2 and CSF1R-CSF1 axes [19]. While the tumor intends to differentiate these recruits into supportive TAMs, CAR-M therapy exploits this mechanism as a “Trojan Horse” strategy. CAR-Ms preserve these chemokine receptors, allowing them to follow the same gradients to penetrate deep into immunologically “cold” tumors [20]. Furthermore, macrophages constitutively express matrix metalloproteinases (MMPs), which enzymatically degrade the dense ECM, effectively “cleaving” a path for their own infiltration and potentially facilitating the subsequent recruitment of ancillary immune effectors [21].

### 2.2. Plasticity as a Therapeutic Lever: Rewiring the TME

**Figure 1:**
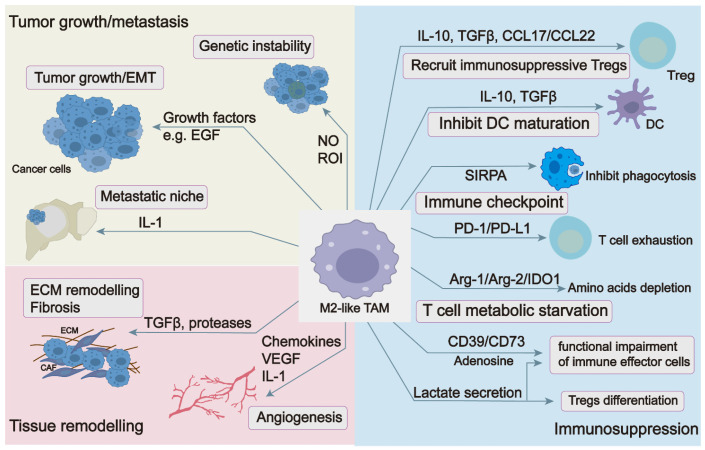
The multifaceted barriers imposed by M2-like Tumor-Associated Macrophages (TAMs). TAMs serve as central architects of the hostile tumor microenvironment (TME), orchestrating both tumor progression and immune exclusion. (Left) Promotion of Tumor Growth and Remodeling: TAMs secrete growth factors (e.g., EGF) and mutagenic species (NO, ROI) to drive cancer cell proliferation and genetic instability. They also release pro-angiogenic factors (VEGF) and proteases (MMPs) to facilitate angiogenesis and ECM remodeling, creating a physical barrier that limits drug and cell penetration. (Right) Orchestration of Immunosuppression: TAMs actively suppress anti-tumor immunity through three key mechanisms: (1) Recruitment and Inhibition: Secreting chemokines (CCL17/22, CXCL12) to recruit Tregs while inhibiting DC maturation via IL-10/TGF-β. (2) Checkpoint Engagement: Expressing PD-L1 and SIRPα to induce T cell exhaustion and evade phagocytosis (“Don’t eat me” signal). (3) Metabolic Sabotage: Depleting essential amino acids via Arg-1/IDO1 and generating immunosuppressive adenosine through the CD39/CD73-mediated hydrolysis of extracellular ATP, collectively rendering effector T cells anergic. These pleiotropic suppressive mechanisms represent the primary targets that CAR-M therapy aims to reprogram or overcome.

Macrophages exhibit remarkable plasticity, existing along a dynamic continuum rather than in fixed binary states [22]. Although the M1/M2 paradigm remains a useful conceptual framework, macrophage activation encompasses a broad spectrum of intermediate and context-dependent phenotypes, including multiple M2-like subtypes (e.g., M2a, M2b, M2c, and M2d) [23,24]. In response to microenvironmental cues, tissue-resident macrophages can acquire pro-inflammatory M1-like characteristics following stimulation with IFN-γ and LPS, or adopt alternatively activated M2-like programs in response to cytokines such as IL-4 and IL-13 [25]. Importantly, these activation states are highly reversible, reflecting the intrinsic plasticity that distinguishes macrophages from terminally differentiated cells [26,27]. In the initial stages of tumorigenesis, the TME is often characterized by moderate hypoxia and an infiltration of immune-stimulatory M1-like macrophages that actively constrain tumor growth [28]. However, as the tumor expands, a pivotal metabolic transition occurs. Cancer cells induce profound acidification via lactate secretion and release specific factors (e.g., CSF-1, TGF-β), which hijack the plastic nature of macrophages. This “metabolic reprogramming” drives a chronological phenotypic shift, converting the anti-tumor M1 population into immunosuppressive M2-like TAMs that dominate advanced tumors, often constituting over 50% of the immune infiltrate [11,29,30]. In addition, accumulating evidence suggests that TAMs can interact with cancer stem cells (CSCs) to support stemness maintenance, tumor progression, and therapeutic resistance. For example, TAM-derived IL-6 can activate STAT3 signaling in CSCs [31], while macrophage–CSC crosstalk has also been shown to promote CSC maintenance through a paracrine EGFR/STAT3/SOX2 signaling axis [32], further highlighting the multifaceted role of macrophages within the tumor microenvironment [33,34]. As illustrated in Figure 1, these M2-like TAMs establish a self-reinforcing immunosuppressive barrier by promoting angiogenesis, suppressing T-cell activity, and aiding metastasis.

**Figure 2:**
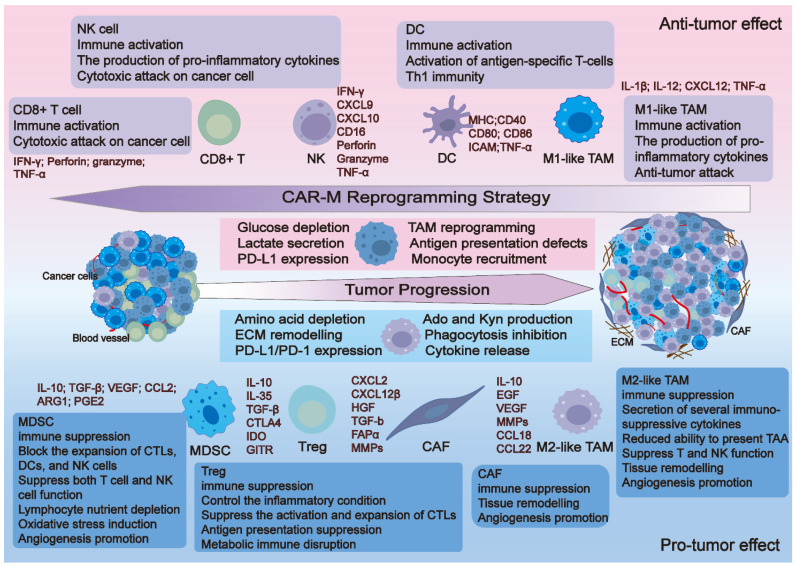
The strategic reversal of the tumor microenvironment (TME) by CAR-M therapy. The TME naturally evolves from an initial immune-active state (top/left) to a terminal immune-suppressive state (bottom/right), driven by cancer cell-secreted factors (e.g., lactate, TGF-β) that polarize macrophages into M2-like TAMs. These TAMs subsequently suppress T cell and NK cell function via checkpoint engagement and metabolic competition. The “CAR-M Reprogramming Strategy” (central arrow) illustrates the therapeutic intervention: by introducing CAR-engineered macrophages, we aim to override these suppressive cues, forcibly reverting the TME phenotype from pro-tumorigenic back to anti-tumorigenic, thereby restoring the cytotoxic potential of endogenous CD8^+^ T cells and NK cells.

While this plasticity fuels tumor progression, it also serves as a therapeutic lever. Crucially, studies have demonstrated that the introduction of CAR signaling domains, including CD3ζ- and FcRγ-based intracellular signaling modules, can reprogram macrophage effector functions and promote antigen-dependent phagocytic activity [20,35]. The signaling activity generated by CAR constructs has been shown to induce and maintain pro-inflammatory macrophage programs, enabling resistance to immunosuppressive cues within the tumor microenvironment [20,36]. Consequently, CAR-Ms can sustain M1-associated cytokine production, enhance antigen presentation, and promote inflammatory remodeling of the TME even under hostile metabolic conditions [20,36,37]. This capacity allows CAR-Ms to function as “microenvironment converters,” shifting the tumor milieu toward an immune-activating state and restoring anti-tumor immune responses (Figure 2) [37].

### 2.3. Bridging Innate and Adaptive Immunity: The APC Advantage

Perhaps the most distinct advantage of CAR-Ms over CAR-T or CAR-NK cells is their function as professional antigen-presenting cells (APCs). CAR-T cells kill targets carrying specific antigens but are powerless against antigen-negative variants, a common cause of relapse. In contrast, CAR-Ms initiate a multi-step immune cascade: (1) Phagocytosis: Upon CAR recognition, CAR-Ms engulf the entire tumor cell. (2) Processing: The tumor cargo is degraded in the lysosome, releasing the CAR-targeted antigen along with a diverse array of tumor-associated antigens (TAAs) and neoantigens. (3) Presentation: These peptides are loaded onto MHC-I and MHC-II molecules and presented to T cells. This process, known as “epitope spreading” [20,38], effectively vaccinates the patient in situ, recruiting and activating the endogenous T cell repertoire against secondary targets that the CAR was not originally designed to recognize. A comprehensive comparison of the biological and functional differences between CAR-T and CAR-M therapies is detailed in Table 1.

## 3. Structural Engineering of Macrophage-Specific CARs

**Figure 3:**
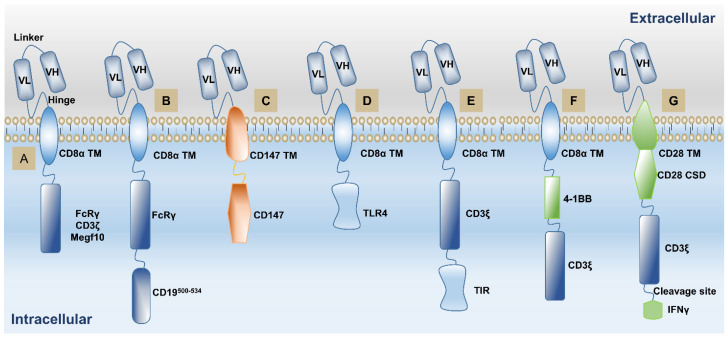
Functional diversity of intracellular signaling domains in CAR-M engineering. (**A**) Phagocytic Signaling Modules. Constructs utilizing canonical ITAM-containing domains, such as CD3ζ, FcRγ, or Megf10. These domains recruit Syk kinase to trigger actin polymerization and cytoskeletal rearrangement, driving direct phagocytosis of tumor cells. (**B**) Tandem Phagocytic Boosters. Fusion of the CD19 cytoplasmic tail (containing the YXXM motif) with FcRγ enhances PI3K recruitment, significantly augmenting the capacity to engulf large cellular targets. (**C**) Matrix-Remodeling Modules. Incorporation of CD147 domains hijacks the matrix metalloproteinase (MMP) regulatory pathway, enabling CAR-Ms to degrade extracellular matrix (ECM) components upon antigen binding. (**D**,**E**) Innate Activation Modules. Integration of the TIR domain from TLR4 (either alone or fused with CD3ζ) activates the NF-κB pathway. This signaling output locks the macrophage in a pro-inflammatory M1 phenotype and stimulates cytokine secretion (e.g., TNF-α, IL-12). (**F**) Persistence Modules. Inclusion of the 4-1BB co-stimulatory domain activates survival pathways (e.g., TRAF2/TRAF1), extending the longevity and persistence of CAR-Ms in the TME. (**G**) Cytokine-Armored Modules. Designed to deliver a payload, these constructs contain a cleavable IFN-γ element. Upon CAR expression/processing, IFN-γ is released to provide autocrine/paracrine support for sustained anti-tumor polarization.

The functional output of a CAR-M—whether it primarily phagocytoses, remodels the matrix, or secretes cytokines—is dictated by its intracellular signaling architecture. Unlike the linear evolution of CAR-T generations, CAR-M engineering follows a modular design principle, where specific domains are selected to address distinct barriers in the solid TME. As summarized in Figure 3, current strategies can be broadly categorized into signaling motifs that drive phagocytosis, polarization, persistence, or matrix remodeling.

### 3.1. Rewiring Phagocytosis

Historically, the CD3ζ chain served as the foundational signaling module. As incorporated in clinical candidates like CT-0508, CD3ζ couples CAR signaling to the endogenous phagocytic machinery via Syk kinase recruitment [20]. However, to optimize engulfment efficiency, researchers have explored alternative myeloid-specific receptors. Morrissey et al. systematically investigated a panel of murine phagocytic receptors and identified intracellular signaling domains capable of promoting antigen-specific phagocytosis by engineered macrophages [42]. Although these findings established the foundational design principles for CAR-mediated phagocytosis, their translational relevance should be interpreted cautiously, as species-specific differences in macrophage biology and tumor microenvironmental interactions may influence therapeutic performance. Subsequent studies using human macrophages have nevertheless provided encouraging validation of these concepts. This library includes receptors such as multiple EGF-like domain protein 10 (Megf10), Fc receptor γ-chain (FcRγ), adhesion G protein-coupled receptor B1 (Bai1), and tyrosine protein kinase Mer (MerTK) [46]. This study revealed that the cytoplasmic domains of Megf10 and FcRγ are sufficient to initiate phagocytosis even in the absence of their native extracellular domains, highlighting their potential as novel signaling modules for CAR engineering [42]. To further empower macrophages to ingest large targets (e.g., whole tumor cells), the “CAR-P” strategy fuses the CD19 cytoplasmic tail (amino acids 500–534, containing the Y_482_XXM motif) to FcRγ [42,47]. This tandem design recruits the p85 subunit of PI3K, a critical driver of membrane extension, thereby tripling the phagocytic capacity compared to single-domain CARs [42,48]. Similarly, Niu et al. demonstrated that integrating the MerTK domain into a C-C chemokine receptor type 7 (CCR7)-targeted CAR mimics endogenous efferocytosis pathways to enhance systemic anti-tumor immunity [49].

### 3.2. Overcoming Physical Barriers

The dense extracellular matrix (ECM) of solid tumors forms a physical fortress that excludes immune cells [50]. To breach this barrier, Zhang et al. engineered CAR-147, which utilizes the intracellular domain of CD147, a regulator of matrix metalloproteinases (MMPs). Upon antigen binding, CAR-147 specifically upregulates MMP-3, -11, and -14 in macrophages. These engineered cells function as “bio-bulldozers,” degrading collagen and laminin to pave the way for T-cell infiltration, a mechanism distinct from direct phagocytosis [51,52]. This autologously produced enzyme arsenal degrades ECM components (collagen/laminin), facilitating T cell infiltration and inhibiting tumor growth [51].

### 3.3. Locking the M1 Phenotype

A major hurdle for CAR-M is the immunosuppressive TME, which promotes M2-like polarization. To counteract this, engineers have integrated innate immune sensors into the CAR architecture: Townsend et al. pioneered the MOTO-CARs™ platform, a novel macrophage-directed chimeric antigen receptor engineered by fusing anti-mesothelin scFv to the intracellular TIR signaling domain of TLR4. This genetic modification significantly enhanced phagocytic activity and augmented the therapeutic efficacy of CAR-M [53]. This construct hijacks the NF-κB pathway, robustly inducing pro-inflammatory cytokines (IL-12, TNF-α) and locking the macrophage in an M1 phenotype [54,55,56]. Lei et al. further validated this by combining CD3ζ with TIR, creating a tandem signaling domain that simultaneously triggers phagocytosis and repolarization [35]. IFN-γ is a potent cytokine that promotes macrophage polarization toward the pro-inflammatory and anti-tumorigenic M1 phenotype [57,58]. Taking this approach, Kang et al. utilized nanocarriers to deliver a plasmid encoding both the CAR and IFN-γ. This “armored” design ensures an autocrine loop where released IFN-γ continuously reinforces the anti-tumor phenotype, preventing M2 reversion in vivo [59]. This approach successfully generated CAR-M1 macrophages, which exhibited potent CAR-mediated cancer cell phagocytosis, modulated the anti-tumor immune response, and consequently suppressed solid tumor growth [59].

### 3.4. Enhancing Persistence

Borrowing from CAR-T logic, the incorporation of co-stimulatory domains can extend CAR-M longevity [60]. Zhang et al. demonstrated that adding 4-1BB (CD137) to the CAR architecture in iPSC-derived macrophages (CAR-iMac) significantly improved survival [61]. Mechanistically, 4-1BB signaling activates TRAF-dependent survival pathways while sustaining the expression of M1 markers (CD80/CD86) and secretion of TNF-α/IL-6 [61].

## 4. The Delivery Vehicle of CAR Molecules

The development of functional CAR-M therapy faces a primary technological bottleneck: achieving efficient, stable, and safe CAR expression within primary macrophages. Unlike T cells, myeloid cells possess robust innate defense mechanisms—such as the SAMHD1 restriction factor and active cytosolic DNA sensors (e.g., cGAS-STING)—that recognize and degrade foreign genetic material [62,63]. Furthermore, their highly active phagolysosomal system efficiently destroys internalized vectors before nuclear entry [64,65]. Consequently, selecting the optimal delivery vehicle is not merely a technical choice but a biological necessity. As summarized in Table 2, current strategies fall into two categories: Viral Vectors (offering stable integration but triggering immune sensing) and Non-Viral Systems (offering transient expression with lower immunogenicity) [66,67].

## 5. Mechanisms of Action: How CAR-M Orchestrates Anti-Tumor Immunity

**Figure 4:**
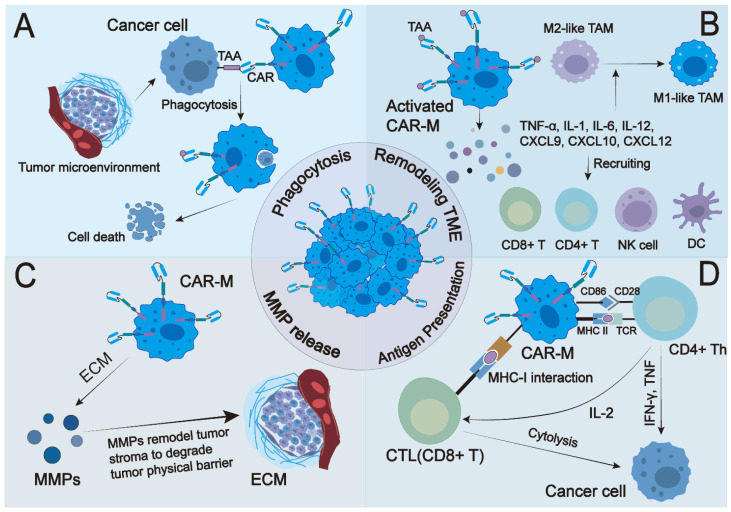
The multifaceted anti-tumor mechanisms of CAR-M therapy. (**A**) Direct Phagocytosis: Upon recognition of tumor-associated antigens (TAAs) via the chimeric receptor, CAR-Ms initiate direct engulfment and lysosomal degradation of cancer cells. (**B**) TME Remodeling: Activated CAR-Ms secrete pro-inflammatory cytokines (e.g., TNF-α, IL-12) and chemokines (e.g., CXCL9/10), which recruit NK cells, T cells, and DCs. Crucially, this inflammatory milieu repolarizes immunosuppressive M2-like TAMs into an anti-tumor M1-like phenotype. (**C**) Matrix Degradation: CAR-Ms release Matrix Metalloproteinases (MMPs) to degrade the dense extracellular matrix (ECM), breaking down the physical barrier to facilitate broad immune infiltration. (**D**) Antigen Presentation and Adaptive Priming: Functioning as professional APCs, CAR-Ms process engulfed neoantigens and present them to CD4^+^ helper T cells (via MHC-II) and CD8^+^ cytotoxic T lymphocytes (CTLs, via MHC-I cross-presentation). Activated CD4^+^ T cells further boost CTL cytotoxicity via IL-2 secretion. Additionally, Th1 cytokines (IFN-γ, TNF-α) secreted by recruited T cells sensitize tumor cells to apoptosis and support B cell-mediated ADCC, culminating in a systemic anti-tumor response.

CAR-M achieves specific antigen recognition via its engineered receptor structure, comprising an extracellular antigen-binding single-chain fragment variable (scFv) domain, a hinge region, a transmembrane domain, and an intracellular signaling domain containing immunoreceptor tyrosine-based activation motifs (ITAMs) [73,74]. Binding of the scFv to tumor-associated antigens, such as human epidermal growth factor receptor 2 (HER2) or mesothelin, induces receptor clustering and ITAM phosphorylation. This activates downstream pathways, including spleen tyrosine kinase (Syk), phosphoinositide 3-kinase (PI3K), and mitogen-activated protein kinase (MAPK) [38,75], driving cytoskeletal reorganization and phagosome formation. Consequently, CAR-M facilitates high-efficiency, antigen-specific phagocytosis of tumor cells [76], leading to their lysosomal degradation [75,77]. This direct physical elimination represents a distinct cytotoxic mechanism that differentiates CAR-M from other cell therapies such as CAR-T cells. As illustrated in Figure 4, this signaling event initiates a multifaceted anti-tumor program that extends far beyond simple cytotoxicity.

### 5.1. Direct Phagocytosis and Lysosomal Clearance

The most immediate consequence of CAR signaling is the cytoskeletal rearrangement that drives antigen-specific phagocytosis. Unlike T cells, which rely on perforin/granzyme-mediated lysis, CAR-Ms physically engulf whole tumor cells or bite off membrane fragments (trogocytosis) [76]. Internalized targets are rapidly trafficked to lysosomes for degradation [75,77]. This mechanism, depicted in Figure 4A, represents a distinct mode of elimination that is effective even against apoptosis-resistant cancer cells.

### 5.2. Epitope Spreading: Bridging Innate and Adaptive Immunity

Perhaps the most critical advantage of CAR-M is its ability to function as a professional antigen-presenting cell (APC). As shown in Figure 4D, following phagocytic degradation, CAR-Ms process tumor-derived peptides and present them not only via MHC-II to CD4^+^ helper T cells but also via MHC-I to CD8^+^ cytotoxic T lymphocytes (CTLs) through cross-presentation [78]. This dual priming is accompanied by the upregulation of co-stimulatory molecules (CD80/CD86). Consequently, CAR-Ms initiate “epitope spreading,” activating the patient’s endogenous T cell repertoire against secondary neoantigens that were not originally targeted by the CAR. This effectively vaccinates the host against tumor heterogeneity and potential antigen escape [59,79].

### 5.3. Remodeling the Hostile TME

CAR-Ms act as potent “microenvironment converters.” The tonic signaling from the CAR construct locks macrophages into a pro-inflammatory M1 phenotype, enabling them to override the immunosuppressive cues of the TME [75]. Polarization to a pro-inflammatory M1 state enables CAR-M to inhibit tumor-promoting M2 macrophages, which are commonly enriched in the TME [80]. Activated CAR-M secretes Th1-polarizing cytokines (e.g., TNF-α, IL-12, and IL-6) and chemokines (e.g., CXCL9/10). While IL-12 and TNF-α directly exhibit anti-tumor effects, they more crucially recruit and activate NK cells and Th1 cells, which in turn secrete high levels of IFN-γ, forming a positive inflammatory feedback loop [75,81,82]. This reversal orchestrates both innate and adaptive anti-tumor immunity. Furthermore, as illustrated in Figure 4C, CAR-M secretes matrix metalloproteinases (MMPs) to degrade the dense ECM. This “stromal remodeling” clears a physical path for infiltrating lymphocytes, addressing a major bottleneck in solid tumor immunotherapy [83,84].

In essence, CAR-M therapy synergistically orchestrates three core mechanisms: (1) direct phagocytic clearance, (2) induction of adaptive immunity via cross-presentation, and (3) inflammatory remodeling of the TME. By integrating direct cytotoxicity with immune modulation, CAR-Ms provide a comprehensive therapeutic platform capable of overcoming the multifaceted barriers of solid tumors.

## 6. The Basic Research of CAR-M for Solid Tumors Treatment

With foundational engineering principles established, the field has rapidly transitioned from in vitro proof-of-concept to rigorous validation in complex immunocompetent animal models. As detailed in Table S1, pioneering studies have now successfully targeted a diverse array of solid tumor antigens—including HER2, Mesothelin, CD19, GD2, and CD147—demonstrating the versatility of the CAR-M platform across varying histologies such as breast, ovarian, pancreatic, and glioblastoma cancers. A critical analysis of these preclinical milestones reveals three distinct evolutionary trends in CAR-M efficacy:

(1) Direct Debulking Capability: Early studies utilizing first-generation CARs (e.g., anti-HER2-CD3ζ) confirmed that CAR-Ms could physically eliminate antigen-positive tumor cells via phagocytosis, leading to significant tumor regression in xenograft models [20,85].
(2) The “Bystander” Awakening: More recent investigations, particularly those using immunocompetent syngeneic models, have highlighted a secondary, perhaps more potent mechanism: the “bystander effect.” For instance, CAR-M treatment was shown to recruit endogenous T cells and repolarize bystander M2-like TAMs, converting the overall TME from “cold” to “hot”. This confirms that CAR-Ms function not just as effectors, but as orchestrators of systemic immunity [86].
(3) Overcoming Anatomical Barriers: Innovative delivery strategies have expanded the reach of CAR-M. Notably, intracavitary delivery of CAR-Ms in glioblastoma models demonstrated the ability to cross anatomical barriers and clear residual glioma stem cells, a feat difficult for systemic therapies [72,87].

Collectively, these preclinical data (summarized in Supplemental Table S1) provide robust evidence that CAR-Ms can overcome the two primary hurdles of solid tumors: antigen heterogeneity (via epitope spreading) and the immunosuppressive stroma (via TME remodeling). These successes have laid the groundwork for the ongoing First-in-Human clinical trials.

## 7. The Clinical Landscape: Early Signals from Phase I Trials

Building on compelling preclinical evidence, CAR-M therapy has entered the crucible of clinical translation. As summarized in Table 3, the landscape is currently dominated by Phase I exploratory trials focused on safety and feasibility.

The most significant finding from early trials, particularly the landmark CT-0508 study (NCT04660929), is the favorable safety profile. Unlike CAR-T therapies, CT-0508 exhibited a favorable and manageable safety profile, characterized by the absence of dose-limiting toxicities (DLTs), high-grade CRS, or neurotoxicity (ICANS), even at high doses. This suggests that CAR-Ms may offer a safer therapeutic window for solid tumors [20].

While complete responses (CR) remain elusive in these early cohorts, encouraging biological activity has been observed. In the CT-0508 trial targeting HER2-overexpressing solid tumors, patients achieved stable disease (SD), and biopsies revealed a crucial mechanistic validation: increased T-cell infiltration and TME remodeling post-treatment [88]. Similarly, the MCY-M11 trial (NCT03608618), utilizing mRNA-electroporated macrophages for mesothelin-positive tumors, demonstrated feasibility, although the transient nature of mRNA expression highlights the need for sustained delivery platforms.

## 8. The Challenges Facing CAR-M Therapy

Despite the unique potential of CAR-M, its transition from bench to bedside is impeded by intrinsic biological and technical barriers. Here, we dissect the three primary bottlenecks: manufacturing scalability, in vivo trafficking, and therapeutic persistence.

### 8.1. The Manufacturing Bottleneck: Cell Source and Expansion

The most immediate hurdle lies in the production of clinical-grade products. Unlike T cells, which can be expanded ~100-fold ex vivo, primary macrophages are terminally differentiated and do not proliferate [89,90,91]. This imposes a strict “input–output” limit: the number of CAR-Ms harvested is directly limited by the number of monocytes collected via leukapheresis. To address this, the selection of the donor cell origin is critical. As summarized in Table 4, current strategies rely on autologous PBMC-derived monocytes, which ensures safety but suffers from donor variability and limited yield. Conversely, iPSC-derived platforms provide a limitless, standardized substrate for the mass production of “off-the-shelf” CAR-M products, bypassing the inherent donor-to-donor variability of PBMCs [10,91]. However, the clinical translation of iPSC-derived CAR-Ms also raises important safety considerations, including the potential presence of residual undifferentiated iPSCs, the risk of unintended genetic alterations introduced during genome engineering, and the need for long-term evaluation of biodistribution and persistence following administration [92,93]. Although THP-1 cells are widely used for preclinical studies and CAR-M engineering research, their leukemic origin precludes their use in clinical applications.

### 8.2. The Trafficking Challenge

Even if high-quality CAR-Ms are produced, delivering them to the tumor site is non-trivial. Following intravenous (i.v.) infusion, systemically administered CAR-Ms are susceptible to sequestration within pulmonary and hepatic microvasculature, a phenomenon termed the “lung–liver trap” that diminishes intratumoral bioavailability [20,59,94]. While locoregional delivery (e.g., intraperitoneal or intracranial injection) can bypass this issue for specific cancers like ovarian cancer or glioblastoma [10,95], it does not address metastatic disease.

Future studies may explore strategies to improve the biodistribution of systemically administered CAR-Ms. One potential approach is the genetic modulation of adhesion molecules involved in pulmonary and hepatic sequestration using genome-engineering technologies such as CRISPR/Cas9, thereby reducing off-target organ retention [40,96]. Mechanistically, circulating monocytes and macrophages can be retained within the pulmonary and hepatic microvasculature through adhesion pathways involving integrins, including Mac-1 (CD11b/CD18) and VLA-4 (α4β1), as well as endothelial adhesion molecules such as ICAM-1 and VCAM-1, which mediate leukocyte arrest, tissue retention, and transendothelial trafficking [18,97]. Selective modulation of these pathways may reduce first-pass sequestration and increase the proportion of CAR-Ms available for tumor homing. However, because many adhesion pathways also contribute to tissue extravasation and tumor infiltration, careful optimization will be required to avoid compromising tumor homing [40]. Alternatively, engineering CAR-Ms to express tumor-relevant chemokine receptors, such as CCR2, CXCR3, CXCR4, or CCR5, may enhance active migration toward chemokine-rich tumor sites and improve intratumoral accumulation [98]. This strategy exploits endogenous chemokine gradients within tumors, including the CCL2–CCR2, CXCL9/10–CXCR3, CXCL12–CXCR4, and CCL5–CCR5 axes, thereby promoting directional trafficking and retention of CAR-Ms within the tumor microenvironment [99,100,101]. In addition, receptor engineering may help overcome the stochastic biodistribution associated with intravenous infusion by providing active navigational cues to target tissues.

### 8.3. Persistence and Phenotypic Stability

A profound functional limitation is the lifespan of the transferred cells. Without self-renewal capacity, CAR-Ms have limited persistence in vivo compared to memory T cells [102]. Furthermore, the plasticity that makes macrophages potent also makes them vulnerable. The TME, rich in TGF-β and IL-10, exerts constant pressure to revert CAR-Ms back to a pro-tumor M2 phenotype [103]. Although engineered receptors (e.g., TLR4-CAR) aim to “lock” the M1 state, long-term phenotypic stability in patients remains a key risk that requires dynamic monitoring [10,104].

### 8.4. Safety Considerations

While early trials suggest a lower risk of neurotoxicity compared to CAR-T, the potential for Cytokine Release Syndrome (CRS) cannot be ignored, given that activated macrophages are the primary source of IL-6 and IL-1 [17]. Additionally, on-target/off-tumor toxicity remains a concern, especially as macrophages naturally reside in healthy tissues.

## 9. Combination Strategies: Orchestrating a Multi-Pronged Attack

### 9.1. Breaking Phagocytic Checkpoints: The “Don’t Eat Me” Axis

The CD47-SIRPα axis is the primary “brake” on macrophage phagocytosis. Even with CAR-mediated activation, the binding of tumor-expressed CD47 to SIRPα on macrophages can blunt the “eat me” signal [105,106]. Strategies to genetically ablate SIRPα in CAR-Ms have shown promise. Sloas et al. used clustered regularly interspaced short palindromic repeats/crispr-associated protein 9 (CRISPR/Cas9) to knock out SIRPα [107], while Zhang et al. utilized shRNA silencing [108]. Both approaches potentiated the phagocytic velocity of CAR-Ms while concurrently engaging the cGAS-STING axis to orchestrate a systemic pro-inflammatory milieu [107,108,109]. In addition to SIRPα blockade, targeting the CD47 molecule on tumor cell surfaces also demonstrated significant synergistic anti-tumor effects. Upton et al. showed that combining anti-CD47 antibodies, such as magrolimab, with trastuzumab (an anti-HER2 antibody) markedly enhanced macrophage-mediated antibody-dependent cellular phagocytosis (ADCP) [110].

As the field moves beyond the well-established CD47-SIRPα axis, novel “Don’t Eat Me” signals are being identified. The CD24-Siglec-10 interaction has emerged as a potent phagocytic brake, particularly in ovarian and breast cancers. While Sun et al. demonstrated the efficacy of blocking this pathway using dual-targeting CAR-T cells in myeloma [111], this strategy offers a direct translational avenue for CAR-M: modifying CAR-Ms to secrete CD24 blockers or designing CARs that target CD24 could unleash phagocytosis in solid tumors [112,113]. Addressing the broader glyco-immune checkpoint landscape, Wu et al. proposed an innovative “tumor-targeted desialylation” approach. By stripping sialic acids from the tumor surface, this strategy disrupts multiple inhibitory interactions (including Siglec-5/10), significantly enhancing the infiltration and activation of CAR-iMacs and converting “cold” tumors into “hot” environments [114]. Furthermore, Li et al. recently identified the Signaling Lymphocytic Activation Molecule (SLAM) family as critical regulators. Deletion of SLAM family receptors (SFRs) was shown to substantially enhance the phagocytic potency of anti-CD19 CAR-Ms, suggesting another genetic engineering target for next-generation products [115].

### 9.2. Releasing the Brakes: Synergy with T-Cell Checkpoints (PD-1/PD-L1)

While PD-1 is famous for T-cell exhaustion, it is also expressed on TAMs, where it correlates with reduced phagocytosis [116]. Blocking the PD-1/PD-L1 axis achieves a dual effect: it reinvigorates exhausted T cells and rescues macrophage phagocytic function [116,117,118]. This rationale underpins the ongoing Phase 1 trial of CT-0508 combined with Pembrolizumab (NCT04660929). Preliminary data suggest that CAR-M treatment upregulates PD-L1 in the TME (an adaptive resistance mechanism), making the addition of a PD-1 inhibitor a logical and necessary step to sustain therapeutic efficacy [20,119].

### 9.3. The “Breach and Strike” Strategy: Combining CAR-M with CAR-T

The dense ECM of solid tumors often traps CAR-T cells at the periphery. CAR-Ms, with their intrinsic MMP secretion and infiltration capacity, can act as “pioneers” to breach this physical barrier [120,121]. In this partnership, CAR-Ms degrade the stroma and recruit T cells via chemokines (CXCL9/10), while CAR-T cells deliver the lethal cytotoxic hit. Shen et al. demonstrated that this combination significantly prolongs survival in murine models compared to either monotherapy [122]. Validating this concept, Klichinsky et al. showed that combining HER2-specific CAR-M with T cells in metastatic SKOV3 models elicited markedly enhanced anti-tumor activity compared to monotherapies [20]. Similarly, Shen et al. demonstrated that the co-infusion of iPSC-derived CAR-Ms and T cells significantly prolonged survival in murine models, highlighting the synergistic potential of this dual-cellular immunotherapy [123]. Beyond physical access, this combination addresses the issue of resistance. Activated CD4^+^ T cells can reprogram intratumoral myeloid cells to kill antigen-loss variants (MHC-I negative cells) that typically evade CD8^+^ CAR-T recognition [124]. Thus, combining CAR-M with CAR-T provides a “failsafe” mechanism: CAR-T cells kill the bulk tumor, while CAR-Ms (and reprogrammed myeloid cells) clean up the antigen-negative variants and remodel the stroma. A recent study identified an indirect mechanism for targeting immune-resistant tumors.

Beyond checkpoint blockade and CAR-T cells, CAR-Ms may also synergize with several emerging therapeutic modalities. As professional antigen-presenting cells, CAR-Ms could enhance the efficacy of cancer vaccines through improved antigen uptake, processing, and cross-presentation, thereby amplifying vaccine-induced T-cell responses [14]. In addition, radiotherapy-induced immunogenic cell death can increase the release of tumor-associated antigens and damage-associated molecular patterns (DAMPs), which may further promote macrophage activation and adaptive immune priming [125,126]. Oncolytic viruses represent another attractive partner, as virus-mediated inflammation and type I interferon signaling can convert immunologically “cold” tumors into “hot” tumors, facilitating immune-cell recruitment and CAR-M activation [127,128]. Conversely, CAR-M-mediated phagocytosis and antigen presentation may enhance antigen spreading and broaden the antitumor immune response initiated by virotherapy. Although these combinatorial approaches remain largely exploratory, they highlight the potential of CAR-Ms to serve as central orchestrators of multimodal cancer immunotherapy.

## 10. Discussion

**Figure 5:**
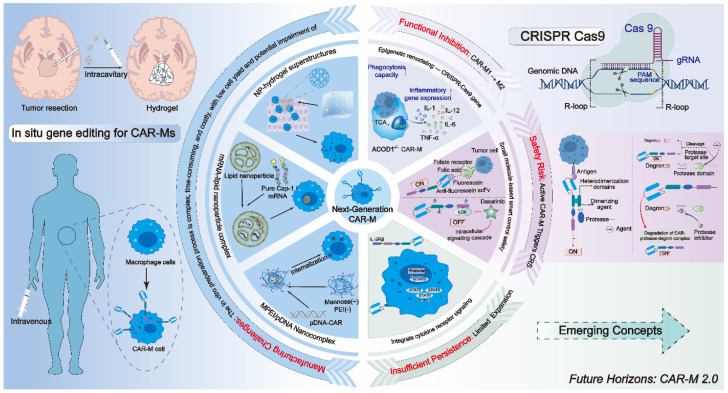
Next-generation strategies to overcome barriers in CAR-M therapy. (Left) Overcoming Manufacturing Bottlenecks: In situ reprogramming strategies utilizing LNP-mRNA complexes (systemic) or Nanoporter-Hydrogels (local/intracavitary) allow for the generation of CAR-Ms directly inside the patient, bypassing ex vivo manufacturing. (Right) Enhancing Safety and Potency: Precision engineering tools such as CRISPR/Cas9 (top right) enable the knockout of inhibitory genes (e.g., ACO1, SIRPA). Logic-gated designs and small-molecule switches provide tunable control over CAR activity to mitigate toxicity risks. The illustrates the integration of cytokine receptor signaling (e.g., IL-2Rb) and metabolic reprogramming modules to sustain M1 polarization and persistence.

As we look beyond the first generation of clinical trials, the field is poised for a technological leap. The next phase of CAR-M development will be characterized by precision control, in situ manufacturing, and synthetic intelligence (Figure 5) [129,130,131,132,133].

### 10.1. Precision Engineering: Metabolic and Logic Gating

Leveraging insights into immunometabolism, researchers are engineering CAR-Ms to resist the hostile TME. For instance, targeting aconitate decarboxylase 1 (ACOD1) has been shown to stabilize the pro-inflammatory phenotype, preventing metabolic exhaustion [134]. Similarly, targeting glycosylation patterns (e.g., desialylation) can disrupt immunosuppressive Siglec signaling, keeping TAMs in an M1 state [135]. To enhance specificity, next-generation constructs are incorporating Boolean logic (AND, OR, NOT). For example, an “AND” gate CAR-M would only activate when it detects both a tumor antigen AND a specific TME signal (e.g., hypoxia), minimizing off-target toxicity [136,137]. Furthermore, novel cytokine-signaling CARs, such as the 28-ΔIL2RB-z (YXXQ) construct, integrate JAK-STAT activation to enhance persistence and prevent exhaustion, marking the advent of “precision-programmable” immunity [138].

### 10.2. The “No-Ex Vivo” Revolution: In Situ Engineering

The high cost and complexity of ex vivo manufacturing remain major barriers. The convergence of synthetic biology and biomaterials is enabling a paradigm shift toward in situ programming. Optimized lipid nanoparticles (LNPs) can deliver CAR-encoding mRNA directly to macrophages in vivo. This “off-the-shelf” approach bypasses the need for leukapheresis and cell culture, turning the patient’s own body into the bioreactor [139,140]. Injectable hydrogels or nanocarriers can create localized “programming centers” within the surgical cavity or TME, ensuring high local concentrations of CAR-Ms while reducing systemic exposure [10,72,141,142].

### 10.3. Drug-Gated Control: The Safety Switch

To address critical safety and efficacy limitations, cutting-edge research now prioritizes “drug-like” controllable systems. Platforms like SNIP (Signal Neutralization by an Inhibitable Protease) and VIPER (Versatile Protease Regulatable CARs) represent a breakthrough, enabling remote, real-time tuning of CAR activity using FDA-approved small molecules [143,144]. These drug-gated circuits provide an on-demand “OFF switch” to manage toxicities like CRS, or a “dimmer switch” to prevent exhaustion, creating antigen density-discriminatory therapeutic windows. The integration of cytokine signaling domains to enhance potency with drug-gated circuits for safety marks the advent of a “precision-programmable” CAR therapy paradigm. Importantly, the fusion of these innovations with iPSC-derived “off-the-shelf” manufacturing has the potential to supplement traditional paradigms, providing universally accessible cellular immunotherapies.

### 10.4. Democratizing Therapy: Off-the-Shelf Platforms

Finally, the transition from autologous to allogeneic products is inevitable [131]. iPSC-derived macrophages (iMacs) offer a scalable, genetically stable source for “off-the-shelf” therapies [145]. Advances in gene editing (e.g., CRISPR/transposons) allow for the precise insertion of complex payloads (CARs + cytokines + safety switches) into safe harbor loci, creating a standardized, universal product that can be deployed rapidly to patients [146,147].

## 11. Conclusions

CAR-M therapy represents a transformative paradigm shift in solid tumor immunotherapy, evolving from a theoretical concept to a clinical reality. By hijacking the innate trafficking and plasticity of macrophages, this modality offers a unique solution to the twin challenges of antigen heterogeneity and the immunosuppressive microenvironment that have long thwarted CAR-T cells. While early-phase clinical trials have established a favorable safety profile and validated the feasibility of manufacturing, the transition from “safety” to “durable efficacy” requires a multidimensional evolution.

The future of CAR-M lies in the transition from simple “phagocytes” to intelligent “microenvironment converters.” This will require next-generation engineering strategies that integrate enhanced persistence, metabolic adaptation, and resistance to tumor-mediated immune suppression. In particular, the CD47–SIRPα and CD24–Siglec-10 axes represent major macrophage immune checkpoints that may limit productive phagocytosis even in the presence of robust CAR signaling. Consequently, combining CAR engineering with anti-phagocytic checkpoint blockade or intrinsic genetic modifications may be essential for maximizing therapeutic efficacy, as these approaches could relieve inhibitory signaling, restore productive engulfment, and enhance downstream antigen presentation and adaptive immune activation [112,148]. Additional priorities include the development of scalable iPSC-derived and in situ engineering platforms, as well as the incorporation of synthetic biology tools such as logic-gated circuits and safety switches.

In summary, the convergence of synthetic biology, immunology, and materials science is actively reshaping CAR-M from a nascent technology into a potent pillar of precision oncology. As these next-generation strategies mature, CAR-M technology is positioned to serve as a pivotal bridge between innate recognition and adaptive priming, redefining the standard-of-care for refractory solid malignancies.

## Figures and Tables

**Table 1:** Comparative Analysis of CAR-T and CAR-M Therapies in Solid Tumors.

Feature	CAR-T Cells	CAR-Ms	Clinical & Translational Implication
Primary Infiltration Mechanism [39,40]	Passive; often physically excluded by dense ECM and aberrant vasculature.	Active recruitment; chemotactically drawn to tumors via CCL2/CCR2 and CSF1R axes.	CAR-Ms possess superior capability to penetrate “cold” solid tumors and dense stroma.
Effector Mechanism [41,42]	Direct cytotoxicity via perforin/granzyme B (MHC-independent).	Phagocytosis (whole-cell engulfment), trogocytosis, and enzymatic ECM degradation (MMPs).	CAR-Ms offer a multi-modal attack, destroying both tumor cells and the physical barrier (stroma).
Antigen Presentation [20]	None (Effector only)	Professional APC; loads tumor peptides onto MHC-I/II complexes.	CAR-Ms induce “Epitope Spreading,” activating endogenous T cells to target heterogeneous antigens, reducing relapse risk.
Response to TME [20,43]	Susceptible to exhaustion (upregulation of PD-1/TIM-3); inhibited by TGF-β.	Susceptible to M2 polarization; however, CAR tonic signaling can lock them in an M1-like inflammatory state.	CAR-Ms act as “Microenvironment Converters,” rewiring the TME from immunosuppressive to immunostimulatory.
Expansion Potential (Manufacturing) [38,41]	High; T cells can expand ex vivo.	Low/None; Terminally differentiated macrophages do not expand.	Major bottleneck: Requires large apheresis volumes or a shift toward iPSC-derived platforms for scalability.
Persistence [20,44]	Long-term (Memory T cells can persist for years).	Limited (Weeks to months); Monocytes naturally turn over.	Requires strategies for repeated dosing or genetic engineering to extend longevity.
Safety Profile (Toxicity) [45]	High risk of CRS (Cytokine Release Syndrome) and ICANS (Neurotoxicity).	Generally lower systemic toxicity; cytokines are produced locally within the TME.	CAR-Ms may offer a safer profile for solid tumor treatment, potentially allowing for diverse dosing schedules.

APC: Antigen-presenting cell; ECM: Extracellular matrix; iPSC: Induced pluripotent stem cell; MMPs: Matrix metalloproteinases; TME: Tumor microenvironment.

**Table 2:** Comparison of Viral and Non-Viral Delivery Systems for CAR-M Engineering.

Delivery System		Design Principle	Efficacy	Refs.
Viral Vector Systems	Lentivirus	Lentivirus co-packaged with Vpx protein (HIV-1 accessory protein)	➢ Chimeric virus exhibited dramatically enhanced infection in human macrophages compared to normal lentivirus ➢ Vpx-containing lentivirus may promote primary human macrophages maintaining in pro-inflammatory state	[68]
	Adenovirus	Adenoviruses with the chimeric type 5 and 35 fiber proteins (Ad5/F35)	➢ The modified Ad5/F35 adenoviral vector to overcome the cell tropism of Ad5 ➢ The elevated transfection efficiency in delivering CAR molecules to primary human macrophages	[20,69]
	Sendai Virus	Non-integrating RNA vector; high transduction efficiency; strong transient gene expression	➢ Limited experience in macrophage engineering ➢ Transient transgene expression ➢ Limited clinical experience in CAR-M manufacturing	[70]
Non-Viral Systems	Lipid nanoparticles	Delivery of anti-CD19 CAR mRNA using lipid nanoparticles	➢ The CAR macrophages (murine) demonstrated significant cytotoxic effects on B lymphoma in vitro	[71]
	MPEI/pCAR-IFN-γ nanocomplexes	Electrostatic interactions between the positive charge of MPEI and the negative charge of pCAR-IFN-γ	➢ The transfection efficiency was lower than that of lentiviral transduction of pCAR-IFN-γ ➢ Increase in IFN-γ expression in M2 BMDMs by nearly 20-fold	[59]
	Nanoporter-hydrogel	CAR gene-laden nanoporter in the hydrogel	➢ Introduce GSC-targeted CAR genes into MΦ nuclei after intra-cavity delivery ➢ Generate CAR-M in mouse models of GBM	[72]

Vpx: Viral protein X; Ad5/F35: Adenovirus serotype 5/35 fiber chimera; CAR: Chimeric antigen receptor; MPEI: Modified polyethylenimine; Nanoporter: Nanoparticle transporter; pCAR-IFN-γ: Plasmid encoding chimeric antigen receptor and interferon-γ; IFN-γ: Interferon-γ; GSC: Glioma stem cell; MΦ: Macrophage; BMDMs: Bone marrow-derived macrophages; GBM: Glioblastoma multiforme.

**Table 3:** Summary of Registered Clinical Trials of CAR-M Therapies.

Product	Sponsor	Trial ID (NCT)	Phase	Status	Target	Targeted-Cancer	Cell Source	Gene Transfer	Key Finding
MCY-M11	MaxCyte Therapeutics	NCT03608618	Phase I	Completed	Mesothelin	Advanced ovarian cancer and peritoneal mesothelioma	PBMC	mRNA transfection	Demonstrated feasibility and tolerability; no DLTs reported; evidence of T-cell recruitment in post-treatment biopsies. TME remodeling observed. Short vein-to-vein time. Intraperitoneal delivery.
CT-0508	Carisma Therapeutics	NCT04660929	Phase I	Active	HER2	Recurrent or metastatic HER2-overexpressing solid tumors	Autologous monocyte-derived macrophages	Adenoviral vector-Ad5f35	TME remodeling observed.
MT-101	Myeloid Therapeutics	NCT05138458	Phase I/II	Recruiting	CD5	Refractory/relapsed T-cell lymphoma	PBMC	mRNA transfection	Short vein-to-vein time.
MAC-001	Macera therapeutics	NCT06224738	Early Phase I	Recruiting	HER2	Advanced gastric cancer with peritoneal metastases	Autologous macrophages	Adenoviral vector	Intraperitoneal delivery.
SY001	Cyagen Biosciences	NCT06562647	N/A		Mesothelin	Advanced ovarian cancer/pancreatic cancer	N/A	N/A	
CARMA-2101	Centre Oscar Lambret	NCT05007379	N/A		HER2	Breast cancer	N/A	N/A	

NCT: National Clinical Trial; PBMC: Peripheral blood mononuclear cell; TME: Tumor microenvironment; CAR-M: Chimeric antigen receptor macrophage; HER2: Human epidermal growth factor receptor 2; CD5: Cluster of differentiation 5; mRNA: Messenger ribonucleic acid; CAR: Chimeric antigen receptor.

**Table 4:** Manufacturing sources of CAR-M.

Types	Manufacturing Process	Advantages	Disadvantages
PBMC	PBMC-CD14^+^ monocyte-(GM-CSF)-Primary macrophage-CAR-M	✔ Collected from real human tissue materials ✔ Retain their primary morphology ✔ More robust inflammatory properties compared to THP-1	➢ Relatively lower yield of monocytes ➢ Difficult to express exogenous genes ➢ Batch-to-batch variability
iPSC	iPSC-CAR-iPSC-CAR-iMac	✔ Unlimited cell source ✔ Apt to genetic manipulation ✔ Easily standardized preparation protocol	➢ Require strict evaluation of differentiation efficiency ➢ Risk of teratoma formation
THP-1	THP1-CAR-THP1-(PMA,vD3,M-CSF)-CAR-M	✔ Attainable ✔ Unlimited proliferation capacity ✔ Easy to transduce exogenous genes	➢ Biologically distinct from primary macrophages ➢ Not feasible for clinical applications
HPSC	HPSC-CAR-HPSC-(arterial EHT)-CAR-M	✔ Generate highly proliferative macrophages ✔ Derived from real human tissue materials ✔ Stronger immune tolerance	➢ Need to induce cell differentiation to generate macrophages ➢ A nonspecific cell-damaging effect associated with CAR editing

CAR-M: Chimeric Antigen Receptor Macrophage; PBMC: Peripheral Blood Mononuclear Cell; CD14: Cluster of Differentiation 14; GM-CSF: Granulocyte-Macrophage Colony-Stimulating Factor; iPSC: Induced Pluripotent Stem Cell; iMac: iPSC-derived Macrophage; THP-1: Human acute monocytic leukemia cell line (standard designation); PMA: Phorbol 12-Myristate 13-Acetate; vD3: 1,25-Dihydroxyvitamin D3; M-CSF: Macrophage Colony-Stimulating Factor; HPSC: Hematopoietic Stem and Progenitor Cell; EHT: Endothelial-to-Hematopoietic Transition.

## Data Availability

Not applicable.

## Supplementary Materials

The following supporting information can be downloaded at: https://www.mdpi.com/article/10.3390/cells15121113/s1; Table S1: The basic research on CAR-M for solid tumor treatment. References [149,150,151,152,153] are cited in the Supplementary Materials.

## Abbreviations

CAR-T	Chimeric antigen receptor T cell
TME	Tumor microenvironment
CAFs	Cancer-associated fibroblasts
TGF-β	Transforming growth factor-β
ECM	Extracellular matrix
PD-1	Programmed death-1
PD-L1	Programmed death-ligand 1
CTLA-4	Cytotoxic T lymphocytes-associated protein 4
TAMs	Tumor-associated macrophages
CEA	Carcinoembryonic antigen
FDA	U.S. Food and Drug Administration
CAR-Ms	CAR-macrophages
CRS	Cytokine release syndrome
M0	Tissue-resident macrophages
IFN-γ	Interferon-γ
LPS	Lipopolysaccharide
IL-4	Interleukin-4
IL-13	Interleukin-13
TLR	Toll-like receptor
TNFα	Tumor necrosis factor-alpha
GM-CSF	Granulocyte-macrophage colony-stimulating factor
CD80	Cluster of differentiation 80
MHC-II	Major histocompatibility complex class II
IFN-γR	Interferon-gamma receptor
iNOS	Inducible nitric oxide synthase
IL-1β	Interleukin-1β
CCL2	C-C motif chemokine ligand 2
CXCL9	C-X-C motif chemokine ligand 9
MyD88	Myeloid differentiation primary response 88
NF-κB	Nuclear factor-kappa B
JAK	Janus kinase
STAT1	Signal transducer and activator of transcription 1
Notch1	Notch homolog 1
IRF5	Interferon regulatory factor 5
Th1	T helper 1 cell
Arg-1	Arginase-1
FIZZ1	Found in inflammatory zone 1
Ym1/2	Chitinase-like protein 3 (Ym1) and chitinase-like protein 4 (Ym2)
PPARγ	Peroxisome proliferator-activated receptor gamma
Syk	Spleen tyrosine kinase
PI3K	Phosphatidylinositol 3-kinase
Smads	Sma and mad related proteins
A2AR	Adenosine A2A receptor
cAMP	Cyclic adenosine monophosphate
PKA	Protein kinase A
HIF-1α	Hypoxia-inducible factor 1 alpha
IL-10	Interleukin-10
CXCL10	C-X-C chemokine ligand 10
CD16	Cluster of differentiation 16
TNF-α	Tumor necrosis factor-α
MHC	Major histocompatibility complex
ICAM	Intercellular adhesion molecule
IL-1b	Interleukin-1β
IL-12	Interleukin-12
CXCL12	C-X-C chemokine ligand 12
TGF-b	Transforming growth factor-β
VEGF	Vascular endothelial growth factor
ARG1	Arginase 1
PGE2	Prostaglandin E2
CTLs	Cytotoxic T lymphocytes
CTLA4	Cytotoxic t-lymphocyte-associated protein 4
IDO	Indoleamine 2,3-dioxygenase
GITR	Glucocorticoid-induced TNF receptor-related protein
HGF	Hepatocyte growth factor
FAPa	Fibroblast activation protein α
MMPs	Matrix metalloproteinases
EGF	Epidermal growth factor
TAA	Tumor-associated antigens
CSF1	Colony-stimulating factor 1
EMT	Epithelial–mesenchymal transition
GPNMB	Glycoprotein nonmetastatic melanoma protein B
Arg-2	Arginase 2
CD39	Ectonucleoside triphosphate diphosphohydrolase-1 (ENTPD1)
CD73	Ecto-5’-nucleotidase (NT5E)
scFv	Single-chain Fragment variable
ITAMs	Immunoreceptor tyrosine-based activation motifs
HER2	Human epidermal growth factor receptor 2
MAPK	Mitogen-activated protein kinase
APCs	Antigen-presenting cells
MHC II	Major histocompatibility complex class II molecules
CTL	Cytotoxic T lymphocyte
IL-1	Interleukin-1
IL-6	Interleukin-6
VH	Variable heavy
VL	Variable light
Megf10	Multiple EGF-like domains protein 10
FcRγ	Fc receptor γ-chain
Bail	Adhesion G protein-coupled receptor B1
MerTK	Tyrosine-protein kinase Mer
CCR7	C-C chemokine receptor type 7
CAR-P	Chimeric antigen receptor for phagocytosis
TLR4	Toll-like receptor 4
TIR	Toll/IL-1 receptor
NF-κB/p65	Nuclear translocation of nuclear factor kappa-light-chain-enhancer of activated B cells- rela
TNF	Tumor necrosis factor
iPSCs	Induced pluripotent stem cells
CAR-iMac	CAR-expressing ipscs-derived macrophage
HLA-DR	Human leukocyte antigen-DR
HIV-1	Human immunodeficiency virus type 1
Ad5/F35	Adenoviruses with the chimeric type 5 and 35 fiber proteins
GBM	Glioblastoma multiforme
PBMCs	Peripheral blood mononuclear cells
THP-1	Human myeloid leukemia mononuclear cells
HSPCs	Hematopoietic stem/progenitor cells
PBMC	Peripheral blood mononuclear cell
iPSC	Induced pluripotent stem cell
PMA	Phorbol-12-myristate-13-acetate
vD3	25-dihydroxyvitamin D3
M-CSF	Macrophage colony-stimulating factor
HPSC	Human pluripotent stem cell
EHT	Endothelial-to-hematopoietic transition.
hFc	Humanized antibody constant region
FcγR1	Fc gamma receptor I
hPSCs	Human pluripotent stem cells
OX40	Tumor necrosis factor receptor superfamily member 4
TM	Transmembrane
CSD	Co-stimulatory domain
BMDMs	Bone marrow-derived macrophages
ALK	Anaplastic lymphoma kinase
NP	Nanoparticles
TNF-α	Tumor necrosis factor-alpha
GSCs	Glioma stem cells
EGFRvIII	Epidermal growth factor receptor variant III
U87MG	U87 malignant glioma
CCL9	Chemokine (C-C Motif) ligand 9
4-1BB	4-1B B lymphocyte antigen
H2030BrM	H2030 brain metastasis
TK1	Thymidine kinase 1
CB-HSPCs	Cord blood-hematopoietic stem and progenitor cells
2B4(CD244)	Cluster of Differentiation 244
DAP12	DNAX-activating protein of 12 kda.
i.v.	Intravenous
i.p.	Intraperitoneal
ICIs	Immune checkpoint inhibitors
SIRPα	Signal regulatory protein alpha
CRISPR/Cas9	Clustered regularly interspaced short palindromic repeats/crispr-associated protein 9
shRNA	Short hairpin ribonucleic acid
ROS	Reactive oxygen species
NO	Nitric oxide
cGAS-STING	Cyclic guanosine adenosine synthase-stimulator of interferon genes
ADCP	Antibody-dependent cellular phagocytosis
SCLC	Small cell lung cancer
BCMA	B-cell maturation antigen
Siglec-10	Sialic acid-binding immunoglobulin-like lectin 10
SFRs	Signaling lymphocytic activation molecule family receptors
ACOD1	Aconitate decarboxylase 1
synBio	Synthetic biology
mRNA	Messenger ribonucleic acid
LNP	Lipid nanoparticle
DAMPs	Damage-associated molecular patterns
ICD	Immunogenic cell death
SNIP	Signal neutralization by an inhibitable protease
VIPER	Versatile protease regulatable
