# Supplementary material for: CAR-M Therapy: From Concept to Clinical Translation in Solid Tumors
Source: Cells. 2026 Jun 19;15(12):1113. doi: 10.3390/cells15121113 (PMC13297294; doi:10.3390/cells15121113)
Supplement: Supplementary file 1 [file cells-15-01113-s001.zip › cells-4288227-supplementary.pdf]

Supplemental Table S1

Targeted-cancer	Cell source	Gene transfer	CAR structure	Results	Refs.
Gastric carcinoma	Monocytes derived from human PBMCs	Adenoviral vector	Anti-**CEA** scfv-hFc-FcγR1	In-vitro results demonstrated potent cytotoxicity against CEA-expressing tumor cell lines;In xenograft studies, targeted monocyte cultures significantly reduced in vivo tumor growth rates and improved survival rates over that of control monocyte cultures.	[15]
Melanoma Neuroblastoma	hPSCs-derived	CRISPR-Cas9	Anti-**GD2** scFv-Hinge-CD28TM-CD28-OX40 CSD-CD3ζ	In vitro, anti-GD2 CAR-Ms significantly enhanced cytotoxic activity against neuroblastoma and melanoma cells;<br>In vivo, anti-GD2 CAR-Ms exhibited potent antitumor efficacy against neuroblastoma (using xenograft mouse models)	[153]
Burkitt lymphoma (Raji B)	J774A.I Macrophages BMDMs	Lentiviral vector	Anti-**CD19** scFv-CD8 TM-Megf10<br>Anti-**CD22** scFv-CD8 TM-Megf10<br>Anti-**CD19** scFv-CD8 TM- FcγR<br>Anti-**CD19** scFv-CD8 TM-CD3ζ<br>Anti-**CD19** scFv-CD8 TM-FcγR-CD19$^{500\text{-}534}$	CAR-P expressing murine macrophages reduce cancer cell number in co-culture by over 40%	[42]
Breast cancer (4T1)	Raw264.7	Lentiviral vector	Anti-**HER2** scFv-IgG1 Hinge-CD147 TM-CD147	In vitro, CAR-147 macrophage treatment did not affect tumour cell growth compared with control treatment.<br>In vivo, CAR-147 macrophages significantly inhibited HER2-4T1 tumour growth, and reduce tumour collagen deposition and promote T-cell infiltration into tumours	[52]

Ovarian cancer<br>Pancreatic cancer<br>Leukemia<br>Lymphoma	iPSCs-derived	Lentiviral vector	Anti-**CD19** scFv-CD8 TM-CD86 CSD-FcγR1<br>Anti-**mesothelin** scFv-CD8 TM-4-1BB CSD-CD3ζ	In-vitro CAR-iMacs showed cytokines expression and phago cytosis in an antigen-dependent manner and polarized them toward an M1 state<br>In-vivo result CAR-iMacs showed some anti-tumor effect on leukemia cells expressing and high mesothelin-expressing ovarian cancer cell line	[61]
N/A	PBMCs	Adenoviral vector Ad5f35	Anti-**HER2** scFv-Hinge-CD3ζ	Anti-HER2 CAR-monocytes produced pro-inflammatory cytokines in response to antigen, specifically phagocytosed HER2 overexpressing target cells, and eradicated HER2-overexpressing tumor cells in a time and dose-dependent manner.	[150]
Ovarian cancer	THP-1	Adenoviral vector (Ad5f35)	Anti-**HER2** scFv-CD8α Hinge- CD8α TM-CD3ζ	In-vitro sustainable M1 phenotype and polarize M2 toward M1 phenotype. CAR-Ms phagocyte and eliminate tumor cells in an antigen-dependent manner.<br>In xenograft mice models, significantly decrease tumor burden and improve overall survival.	[20]
Lung tumors	PBMCs	Adenoviral vector (Ad5f35)	Anti-**mesothelin** scFv-CD8 Hinge- CD8 TM-CD3ζ	In-vitro: high CAR expression, possesses M1 phenotype with relative resistance to M2 shift, and exhibits robust tumor cell-killing capability and pro-inflammatory cytokines;<br>In vivo, CT-1119 significantly reduced tumor burden in a murine xenograft model of lung cancer.	[85]
Neuroblastoma	Macrophages in situ	Nano complex jetPEI macrophage(MPEI)	Anti- **ALK** scFv- CD8 Hinge- CD28 TM-CD28 CSD-CD3ζ-IFN-γ	The MPEI/pCAR-IFN-γ injection into Neuro-2a tumor-bearing mice reduced tumor growth, decreased the Treg cell, and increased the function of activated $CD8^+$ T cells in the tumors.	[59]

Glioblastoma multiforme	Macrophages in situ	NP-hydrogel	Anti-**CD133** scFv -Hinge-CD3ζ	In-vitro NP-pCAR induces M1 polarization and increases the secretion of IL-1β and TNF-α of targeted macrophages.<br>In the orthotropic mouse glioma model, CAR-Ms could seek and engulf GSCs and clear residual GSCs by stimulating an adaptive antitumor immune response.	[72]
Brain stem glioma	Macrophages in situ	Nano complex	Anti-**HER2** CAR-Ms	In-vitro: nanoparticles produce CAR-Ms with M1 phenotype and greater phagocytic and cytotoxic ability in an antigen-dependent manner;<br>By acting as a "living" cure, the HER2-specific CAR-Ms that were produced tracked and devoured cancerous cells and serially initiated innate and adaptive anti-antitumor responses to facilitate reversal of the immunosuppressive TME	[87]
B lymphoma	RAW264.7 BMDMs	Lipid nanoparticle	Anti-**CD19** scFv -Hinge-CD3ζ	In vitro, the CAR-Ms demonstrated significant cytotoxic effects on B lymphoma.	[71]
EGFRvIII positive cells (U87MG cells)	IPSCs-derived	Lentiviral	Anti-**EGFRvⅢ** scFv-CD8α TM- CD3ζ-TIR	CD3ζ-TIR-CAR, the second generation of TIR-based dual signaling CAR endowed iMACs the target engulfing capacity against antigen-expressing tumor cells, as well as potency of antigen-dependent M1 polarization and resistance to M2 polarization in a NF-κB dependent manner.	[151]
Burkitt lymphoma (Raji B)	BMDMs	Lentiviral	Anti-**CD19** scFv CD8α Hinge/TM-FcRγ<br>Anti-**CD19** scFv CD8α Hinge/TM-PI3K<br>Anti-**CD19** scFv CD8α Hinge/TM-Megf10	CAR-M-FcRγ exerted more potent phagocytic and tumor-killing capacity than CAR-M-Megf10 and CAR-M-PI3K.<br>CAR-M and CAR-T demonstrated synergistic cytotoxicity against tumor cells in vitro.	[122]

Breast cancer (4T1)	RAW264.7-derived	Lentiviral	(Anti-**CCR7**)<br>CCL9-CD8α Hinge-TM-TLR2<br>CCL9-CD8α Hinge-TM-TLR4<br>CCL9-CD8α Hinge-TM-TLR6<br>CCL9-CD8α Hinge-TM-MerTK<br>CCL9-CD8α Hinge-TM-4-1BB+CD3ζ	In vitro and in vivo, MerTK-CAR-M demonstrates potent anti-tumor efficacy, exhibiting significantly enhanced phagocytic activity against tumor cells.<br>In vivo, this therapy robustly reduces tumor burden, extends median survival time, and establishes a pro-inflammatory microenvironment through upregulation of serum pro-inflammatory cytokines	[49]
Lung cancer brain metastasis cell (H2030BrM)	N/A	N/A	Anti- **mesothelin** CAR-M with MyD88 signaling molecule	In the humanized mouse model, CAR-Ms penetrated BBB and significantly reduced brain metastasis growth. The CAR-Ms exhibit antigen-specific phagocytosis activity against MSLN-positive tumor cells. Also, CAR-M demonstrates much fewer neuron toxicities and liver compared to CAR-T	[152]
Mammary gland squamous carcinoma cell line (HCC-1806)	RAW 264.7 derived	N/A	Anti- **mesothelin** CAR-M with TLR4 and TLR2-based toll-like receptor signaling domains	In-vitro, MOTO-CARs effectively kill cancer cells and secrete notable levels of TNF-α, which displays that MOTO-CARs polarize toward the M1 phenotype upon target recognition.<br>In-vivo, the MOTO-CARs can traffic effectively to the tumor site and substantially reduce tumor burden compared to the mock control	[53]

Non-small cell lung carcinoma NCI-H460 and A549 cell lines	Human monocyte - derived	lentiviral or adenoviral (Ad5f35)	Anti-**TK1** CAR M with TIR signaling	MOTO-CAR cells showed a consistent M1 phenotype expressing high levels of CD14, CD80, CD206 and low levels of CD163.	[86]
HT1080 cells	CB-HSPCs	Lentiviral vector	Anti-**CEA** scFv-IgG1 Hinge- CD28 TM/CSD-CD3ζ Anti-**CEA** scFv-IgG1 Hinge- 2B4 TM/CSD-DAP12	The engineered macrophages displayed distinct antigen-specific functions depending on the CAR construct: those with anti-CEA-CARs showed increased cytokine secretion, while those expressing the CD3ζ CAR exhibited enhanced phagocytosis of target cells.	[153]
